# Letter from Professor Slack, to Professor James Taylor, M. D.

**Published:** 1848-01

**Authors:** 


### ARTICLE VIII.

*Letter from* Professor SLACK, to JAMES TAYLOR, M. D., D. D. S., Professor of Practical Dentistry and Pharmacy, in the Ohio College, of Dental Surgery, Cincinnati.

DEAR SIR:

I perceive, by the February No. of the "New York Dental Recorder," which you were so good as to allow me to read, that a considerable controversy has been raised in New York, by the action of a dental society, in regard to a quack and most reprehensible method of filling carious teeth with an amalgam of gold or silver, and we presume, more frequently, tin or lead.

I would here remark, that it behoves us, who live far over the mountains, to see that our practice in the only dental school in the west, be regulated by sound principles; let others do as they may.

In an examination of the subject before us, it becomes, in the first place, to attend to a few elementary particulars, which some may call small things, but to my mind, are, at the very foundation of the investigation.

The composition of the tooth, consisting of more than one-third *cellular tissue*, one-half *phosphate of lime*, one-tenth *carbonate of lime*, and a small fraction of fluoride of calcium, is an item to be remembered. The enamel differs from the body of the tooth only in the amount of phosphate of lime, which, on careful decomposition, is found to be 75 per cent.

It is a fact tolerably well decided, that the *silicious coating* of the wheat straw, Indian corn stalk, various grasses—of the cane, ratan, bamboo, &c., is *reticulated.* Also, that the enamel of various oceanic and fluviatile shells possess the same form of structure. The microscope examination of the exterior of a well conditioned tooth leads to the same result, i. e. a smooth reticulated or net work coating.

Secondly. Eremacansis is *slow combustion* or *gradual decay*, and has been ably investigated by Liebig, and confirmed by Dumas Gregory, and all chemists, who endeavor to keep pace with the rapid march of this noble science. This process of decay, slow combustion or oxydation, so important to the *dental practitioner* requires certain conditions to promote it. There must be moisture; a certain temperature above 32°, a blood heat 98°; or even higher, as in hot tea or coffee, or other heated articles of diet. There must be oxygen either from the atmosphere, or some other source, for eremacansis decay, or slow oxydation cannot proceed without this *latter*, which the atmosphere must, in 99 cases out of 100 furnish. Decay, or slow oxydation, then must begin in the external part of the tooth. In the interior oxygen could not be furnished, unless by some potent disease a passage be opened to the atmosphere. Eremacansis is the action of a substance in a state of decomposition, *the particles of which are in intestine motion*—placed in contact with another substance, whose chemical affinities are easily overturned, so as to produce the same change in the latter substance: ego, suppose decaying *matter*, with the circumstance above detailed, be brought in contact with the external of a promising tooth, and continued for some time, what, in the living tooth is likely to be first affected? I have already spoken of the reticulated form of the enamel and of the cellular tissue, which contains nitrogen, and which all chemists admit to be held by the feeblest affinities. The substance in decomposition operates, in my view, first, on the nitrogen of the cellular tissue through the small openings of the enamel, and thus produces the same motion and decomposition in a fraction of the living tooth's tissue. I am aware that sometimes the caries seem to attack the phosphate of lime, while the tissue remains, but careful examination, will, I am persuaded, from the very first, disclose in all such cases, a decidedly unhealthy state in the tissue, though the greatest ravage may appear in the phosphate salt. It is my design to be concise, if practicable; and must thirdly consider the chemical action of substances employed in filling carious teeth on the organ in question, and on the general system. I have no objection to the employment of one metal, such as gold, silver, or platina, if in the state of delicate foil. I will not object to tin foil, if it be pure or free from lead, with which it is, in 9 cases out of 10, adulterated in the tin foil of commerce.

The filling ought always to be with one metal, and only one, to avoid galvanic action, which will result where two metals are employed together. A certain amount of electricity is incident to every substance, whether organic or the contrary, and this is necessary in the organised to a healthy condition; but augment the amount and you invariably have unnatural action, which, if continued, will end in certain, and sometimes, fearful disease.

It may be said there are few metals which produce galvanic action. I deny the position, and my denial is confirmed by the gold plate and solder producing galvanic salivation, as reported in the February No. of the "New York Dental Recorder."

Tin and copper vessels always are oxydizing and corroding, where the solder join the parts furnish a decided effect, produced by small quantities of galvanic electricity The principle of delicate galvanic action is shown in Davy's beautiful contrivance for preserving the copper sheeting of vessels in oceanic waters, by *small substratum* of zinc or iron nails—changing by *induction* the electrical state of the copper. On the same *law* a copper kettle in boiling cider or pickles, is rendered measurably innoxious by immersing in the liquid a piece of pure metallic tin; without the tin it will be most certainly poisonous. The law of induction is illustrated by the practice of packing razors or surgical instruments in zinc cases to prevent rust; the electrical state of the steel instruments is changed to the same with the oxygen of the air; of course, union does not take place —even animal matter, as slices of brain and muscle, and vegetable—as sections of beet root and walnut tree furnish an example of distinct galvanic action where no metal is concerned —but to return to the amalgáms noticed above, as used for filling carious teeth. Is it not true that the mercury miners at Idria in Austria, Almeden in Spain, or Guaneavilica in Peru, are frequently affected injuriously by the crude article in which they work? Is it not true that sailors carrying cargoes of metallic mercury are sometimes salivated? These facts show that mercury is easily changed so as to produce the effects noticed; I say changed, for few pure metals are poisonous. Copper, while clean, is inert, but let it be oxydized, i. e. changed, and it becomes poisonous. Lead, when pure, I may affirm, is innoxious, but let it be oxydized, which it must be before combining with carbonic acid, and it is changed; carbonate of lead results, which produces painter's colic, and many other forms of poisoning.

Amalgams are but another name for alloys. In alloys it is a principle that the compound is more changeable than the simples forming the combination. Alloys universally attract oxygen more energetically than the metals of which they are composed. Is not an alloy more inclined to oxydation, i. e. to chemical action, and of course, to galvanic action than its simples?

Does not galvanic action, even in the common battery, primarily result from the oxydation of one metal in the series? Is not this the laws of the battery? Mercury and gold, mercury and silver, mercury and tin, are peculiar combinations or alloys, called amalgams, and these are most strikingly sensible to oxydation, and is not this, of itself, prima facie evidence that galvanic action, to no small extent, results. I speak theoretically, but I have little doubt of the fact, that a few combinations of an amalgam of gold, or silver, or of tin, (I will not mention bismuth or lead) arranged in order, would produce the effects of an imperfect battery. Is it not true, that an amalgam of zinc, tin and mercury (though the tin may be omitted) is considered necessary to the full excitation of the common electrical machine on which the friction of the amalgamed rubber is employed. I admit, with Davy, that the action of this amalgam is unexplained, but I have often thought that possibly the metals combined evolve by friction, galvanic electricity, which is so identical with that of the common glass machines that from this source and the neighboring bodies a large supply of electricity is regularly furnished. This is not more extraordinary than other facts. I have known considerable electricity collected from a piece of dry wood, moved swiftly in a turning lathe, another dry stick being applied to the former, to maintain the friction. Fire is easily produced by such friction. Hence Count Rumford's *wind-fall* of continued friction, producing caloric as long as the motion was kept up, is not so strange after all. In the new hydro-electrical machine, the wonderful evolution of the electrical fluid, from which sparks 20 inches long were drawn by Faraday, he inferred arose from the friction of the steam against the orifices of the tubes from which it was issuing.

The examples of the electrical amalgam, the dyed wood, and the hydro-electrical machine, are introduced to show the wonderful variety and delicacy in electrical action, which no philosopher has satisfactorily explained. Why may not galvanic action be promoted by friction as well as acids? How do we know that an amalgamed rubber *mechanically* pumps up the electrical fluid? The accumulation of the fluid is known, but the why and wherefore we know not. The delicate galvanometer is affected by the movement of the hand on the slightest friction, clearly showing that the electrical or galvanic fluid is present. If a plate of zinc and a plate of copper, each with an insulating handle, be brought together and suddenly separated by a delicate electromiter, the zinc will be found in a positive state and the copper in a negative. Without this expedient they are both said to be positive. Whence the change? It will be answered by *induction.* Then one plate before the contact must have held more of the fluid than the other. May not the simple contact, producing friction, have generated or excited galvanic action? The subject is curious and recondite, but not probed to the bottom. It may be (a supposition) that friction on the amalgamed rubber, the dry wood, or in the hydro-electrical machine, is one means of promoting galvanic action.

The galvanic and electrical fluids are for the most part identical, except in the common machine, the fluid is more dense, in the other more rare, but in larger quantity. They both decompose; they both effect the living subject with as much similarity as their different circumstances and modifications permit. Galvanic action is so variant that it becomes the dentist to look well to its *laws*, if he expect to excel in his profession, or be, as he professes, a benefactor of mankind.

Again, the amalgams so extensively employed in filling carious teeth before employed in such a service, ought to be subjected to the most rigid chemical examination, that the beauty of the face and the general health of the patient may be promoted—if these important points be not reached, the practice ought to be, must be abandoned, as wholly improper. If eremacansis be promoted by the absorption or action of oxygen—a chemical principle well established—I say, theoretically, but, as I believe, truly, the usual amalgam fillings above noticed, must promote decay. And in Dr. Allen's communication referring to the practice, which he seems somewhat to condemn, the fact is abundantly confirmed; and on inquiry, I find a considerable amount of facts, in the west, fully establishing my previous reasonings.

I laid it down above, as a principle somewhat certainly established, that a pure metal, with few exceptions, is innoxious; but one, or especially a compound, easily attracting oxygen, is, in general, deleterious. Will not this remark, based on principles apply most pointedly to the practice in question?

Again: The cellular tissue of the tooth is filled with minute arteries, veins, absorbents and nerves;—leaving out of view galvanic actions—what must be the effect from the oxydation of the inserted amalgam? Will not the deleterious *ide* compound be absorbed? And can a substance certainly poisonous, be any advantage to nerve, artery, veins, blood, &c.? It will be thrown into the circulation, and though the poison may move slowly at first, it will most certainly, unless arrested, perform its work of destruction. Health must be prostrated, and an early grave will be the portion of the victim. Let chemists of experience, or physiologists who understand the chemistry of the system, (which the great Liebig unfolded, and which by others has been most triumphantly confirmed,) speak out, and they will, for the most part, confirm what I have advanced. No other rational conclusion can be drawn from the premises.—*Dental Register.*

